# Association between healthy lifestyle on life course and multimorbidity in adults: results from two national prospective cohort studies

**DOI:** 10.1186/s12889-024-20443-7

**Published:** 2024-10-23

**Authors:** Xiaoying Ye, Mengdan Liang, Zhehui Chen, Xiannuan Jiang, Mengying Xie, Xiaowei Xie, Guohui Lan, Xiaoli Lu, Zelin Huang, Tingting Xu, Xiaoxu Xie

**Affiliations:** 1 Department of Epidemiology and Health Statistics, School of Public Health, Fujian Medical University, Fuzhou, China; 2 The Second Clinical Medical School, Nanchang University, Nanchang, China; 3 The First Clinical Medical School, Shanxi Medical University, Taiyuan, China; 4 Clinical Research Unit, The Second Affiliated Hospital, Fujian Medical University, Quanzhou, China

**Keywords:** Multimorbidity, SCORE2, Life’s essential 8, Healthy lifestyle, Trajectory

## Abstract

**Objectives:**

To examine the correlation between healthy lifestyle patterns, their change trajectories, and the risk of multimorbidity in adults.

**Methods:**

Based on two representative national cohorts, the English Longitudinal Study of Aging (ELSA) and the Health and Retirement Study (HRS) including adults aged 50 years and over. We employed Cox regression, lifestyle change trajectories, and restricted mean survival times to explore the relationship between lifestyle (assessed by SCORE2, LE’8, and HLS scores) and multimorbidity. We also conducted mediation analysis to investigate the underlying mechanisms.

**Results:**

A healthy lifestyle (higher LE’8, higher HLS, or lower SCORE2) can reduce the risk of multimorbidity. 2-10% lower multimorbidity risk per one-point increase in LE’8 and HLS. The hazard ratio of multimorbidity for improvements in unhealthy lifestyles or deterioration in healthy lifestyles compared to always healthy lifestyles ranged from 1.598 to 5.602. Besides, for LE’8 and HLS, participants with higher scores had a slower decrease in survival probability in ELSA. Triglyceride, C-reaction protein, fibrinogen, and cystatin C partly mediate the association between lifestyle and multimorbidity.

**Conclusions:**

Keeping a healthy lifestyle over time can help reduce the risk of multimorbidity.

**Supplementary Information:**

The online version contains supplementary material available at 10.1186/s12889-024-20443-7.

## Key messages

1. The first study to investigate the link of healthy lifestyle patterns and their change trajectories to multimorbidity.

2. Adherence to a healthy lifestyle has a greater disease-preventive effect in the long term than in the near term.

3. Deterioration in healthy lifestyle is associated with a higher risk of multimorbidity.

**Supplementary Information:**

The online version contains supplementary material available at 10.1186/s12889-024-20443-7.

## Key points

**Question** Is lifestyle and its trajectory related to multimorbidity?

**Findings** We found that adherence to healthy lifestyles (higher LE’8 or HLS, lower SCORE2) was associated with a lower risk of multimorbidity. Deterioration in healthy lifestyle is associated with a higher risk of multimorbidity compared to a consistently healthy lifestyle. In addition, adherence to a healthy lifestyle has a greater disease-preventive effect in the long term than in the near term.

**Meaning** Our research findings suggested that a healthy lifestyle can help reduce the risk of developing multimorbidity. The more persistent, the better the quality of life.

**Supplementary Information:**

The online version contains supplementary material available at 10.1186/s12889-024-20443-7.

## Introduction

Chronic non-communicable diseases are implicated in unequal health outcomes, lower quality of life and higher use of health resources. Cancer, cardiovascular diseases (CVD), diabetes and chronic respiratory diseases account for the highest rates of mortality, morbidity, and disability [1]. The largest absolute increases in number of disability-adjusted life years in older adults from 1990 to 2019 also included ischemic heart disease, diabetes, stroke, chronic kidney disease, and lung cancer [2]. However, diseases do not exist independently, the large increase in global burden of stroke was due to the substantial increase in high fasting plasma glucose, high systolic blood pressure [3]. Adverse health outcomes of multimorbidity (two or more coexisting conditions in an individual) [4] are more clinically challenging than any single disease [5]. Multimorbidity is strongly age-related, with prevalence rates ranging from 65 to 82% in people over 65 years of age [1]. At the same time, studies have shown that modifiable lifestyle factors are relevant to reducing the mortality risk of chronic diseases [6–8] and a reduced risk of cardiometabolic multimorbidity [9, 10]. Therefore, with the intensification of aging, attention to reducing multimorbidity in the population by improving lifestyles was deemed warranted.

Healthy lifestyle factors comprise maintaining a nutritious diet, engaging in regular physical exercise, managing body weight, abstaining from smoking and alcohol consumption, and ensuring adequate sleep. Combining multiple healthy lifestyle choices has a greater impact on overall health compared to any single lifestyle factor [11]. In 2020, the American Heart Association released an updated version of its cardiovascular health assessment tool, Life Essentials 8 (LE’8) [12]. Meanwhile, in 2021, the European Society of Cardiology introduced Systematic Coronary Risk Evaluation 2 (SCORE2), an algorithm for estimating the 10-year risk of first-onset cardiovascular disease in European populations [13]. The Healthy Lifestyle Score (HLS) is based on five lifestyle factors (i.e., smoking, alcohol consumption, physical activity, diet, and obesity), which were developed based on a Chinese population [14]. These lifestyle scores are linked not only to cardiovascular health [15], but also to various other conditions. A high cardiovascular health lifestyle score, as defined by LE’8, is linked to a decreased likelihood of stroke, cardiovascular disease, or mortality [16, 17]. SCORE2 is also effective in forecasting dementia and all-cause mortality in European populations ^18^, and it has good predictive value in identifying physical decline among non-frail adults aged 50 and older [19]. However, there is a paucity of definitive research on the relationship between various healthy lifestyles and multimorbidity. Clarifying the relationship between healthy lifestyle and multimorbidity provides reference value for promoting group health and the economic burden of chronic diseases. It provides methods for disease prevention and control strategies, which can be intervened by promoting healthy lifestyles. Besides, due to the changeability of healthy lifestyle behaviors, it was necessary to further explore the impact of lifestyle changes on multimorbidity but remained unclear. In addition, it was worth exploring the short-term and long-term effects of lifestyle on multimorbidity, which was beneficial for policy choices.

Therefore, based on a hypothesis, good health behaviors promote well-being, while poor health behaviors have detrimental effects on health. The purpose of this study is to compare the correlation between healthy lifestyle patterns assessed by SCORE2, LE’8, and HLS scores to the risk of multimorbidity. Additionally, the study will determine the impact of lifestyle change trajectory patterns on multimorbidity utilizing the latent class trajectory model (LCTM). We also evaluated the impact of lifestyle on multimorbidity in the short and long term. The study analyzed data from the English Longitudinal Study of Aging (ELSA) and the Health and Retirement Study (HRS) to account for the population’s diversity and ensure a comprehensive analysis.

## Methods

### Study population

The study population comprised individuals from two nationally representative cohorts: the UK-based English Longitudinal Study of Ageing (ELSA) and the US-based Health and Retirement Study (HRS), both including adults aged 50 years and over. Our study relied on 14-year longitudinal data, spanning from Wave 2004 to Wave 2018 for the ELSA cohort and from Wave 2006 to Wave 2020 for the HRS cohort. The baseline was set for the ELSA cohort at Wave 2004 and for the HRS cohort at Waves 2006 and 2008. After excluding populations without exposed information, baseline patients with 2 or more poorly diseases, and those who were lost to follow-up, the study population ranged from 1156 to 3299. More details of participant exclusion can be seen in eFigure 1 in Supplement. The ELSA study received ratification from the London Multi-Center Research Ethics Committee (MREC/01/2/91), while the HRS study received approval from the University of Michigan Institutional Review Board and the National Institute on Aging (HUM00061128).

### Exposure assessment

Three healthy lifestyle scores, systemic coronary risk estimation 2 (SCORE2), life’s essential 8 (LE’8), and healthy lifestyle score (HLS), were used as indicators of exposure levels. The SCORE2 was a new algorithm for estimating the risk of developing CVD for the first time within 10 years in a European population aged 40–69 years without a prior history of CVD [13]. The risk variables included sex, age, smoking, systolic blood pressure (SBP), total cholesterol (TC) and high-density lipoprotein (HDL). It has been classically categorized in low (1–4%), medium (5–9%), high (> 10%) risk categories. The specific algorithm of SCORE2 was shown in Supplementary methods eTable 1.

The eight indicators of LE’8 is divided into two areas: health behaviors (diet, physical activity (PA), smoking, and sleep) and health factors (body mass index (BMI), non-high-density lipoprotein cholesterol, fasting blood glucose, and blood pressure). Each indicator is scored on a scale from 0 to 100, which then produces a new total cardiovascular health score (an unweighted average of all components) that also ranges from 0 to 100. A total score of 80 to 100 for CVH is considered high; 50 ∼ 79, medium CVH; 0–49 points, low CVH. Due to the lack of dietary and sleep investigation in the ELSA and HRS, only 6 indicators were included in LE8 of this study. The specific algorithm of LE’8 was shown in Supplementary methods eTable 2.

Based on previous study [14], the HLS was built on five lifestyle elements, including smoking, drinking, PA, sleep, and obesity. Each lifestyle element was divided into healthy and unhealthy groups. The unhealthy group was defined, respectively, as people who smoke, drank alcohol, do not vigorous exercise or activity, self-reported poor sleep quality, and obesity (the BMI of < 18.5 or > 23.9 kg/m^2^, and a waistline of > 90 centimeters for men and > 85 centimeters for women). The HLS equals the sum of the number of factors defining the healthy group, varying from 0 (the unhealthiest) to 5 (the healthiest). It also has been classified into three categories: low (0–1), medium (2–3), and high (4–5).

### Outcome

The outcome of this study was self-reported multimorbidity during a biennial follow-up survey. Participants with 2 or more of the following chronic diseases were categorized as multimorbidity: hypertension, diabetes, cancer, chronic lung disease, heart disease, and stroke.

### Statistical analysis

The flowchart of the statistical analysis was showed in Supplementary methods eFigure 1. Baseline information was described, with results shown as numbers (percentages) for discrete variables and medians (interquartile range [IQR]) for continuous variables.

Longitudinal associations between healthy lifestyle scores, estimated by three modalities, namely SCORE2, LE’8, and HLS, and multimorbidity were analyzed using Cox regression, with follow-up time as the time scale. The Schoenfeld test was used to test the proportional hazards assumption. In addition, three lifestyle scores were analyzed as categorical variables. SCORE2 had been classically categorized in low (1–4%), medium (5–9%), high (> 10%) risk categories. A total score of 80 to 100 for LE’8 was considered high; 50 ∼ 79, medium CVH; 0–49 points, low CVH. HLS also had been classified into three categories: low (0–1), medium (2–3), and high (4–5). The marginal effects model was used to explore the association between three lifestyle scores and multimorbidity. Covariates included smoke (yes or no), BMI (continuous variable), age (continuous variable) and race (white, non-white, and missing). In ELSA, the question about drinking was set to “last year’s alcohol intake”, which has the option “no”, “more than once a year”, “more than once a month”, “more than once a week”, “almost every day”, “missing”. In HRS, it was set to “alcohol consumption or not”. The missing continuous variables were interpolated using means. The missing categorical variables were treated as missing indicators in analysis. SCORE2 model was adjusted for drinking, race, and BMI; LE’8 model was adjusted for age, sex, drinking, and race; HLS model was adjusted for age, sex, and race. We calculated *E*-values and False discovery rate (FDR) to strengthen the confidence of the results. FDR < 0.05 was considered statistically significant.

It was found that some models in the ELSA did not conform to the proportional hazards assumption, so the restricted mean survival time (RMST) analysis was carried out. The RMST was the average survival time truncated at a fix point in time, that is, the survival area under the curve at the point in time of the truncation. On the other hand, we use the latent class trajectory model (LCTM) to identify the trajectory of the three lifestyle changes over time. This is a special form of a finite mixture model designed to identify potential classes of individuals with similar progression over time or in determinants of age change. We estimated the most suitable number of tracks based on the posterior probability of class (> 0.70) and class size (≥ 2% population), as well as the minimum Bayesian information criterion. We also conducted mediation analysis to investigate the underlying mechanisms.

Finally, sensitivity analyses were performed to assess the robustness of main results: (1) Apply the database of this study to perform intercept correction on the SCORE2 model and verify the robustness of the results; (2) To avoid reverse causality, the analysis was limited to participants with multimorbidity who were followed for > 2 years; (3) Inverse probability weighting was performed on the three lifestyle scores; (4) We defined mortality as a competitive event to analyze the competing risk model. Bilateral *P* values were used, and *P* < 0.05 was considered statistically significant. All analyses were performed using R version 4.2.3.

## Results

### Descriptive statistics

The baseline characteristics of participants and three lifestyle scores were shown in Table 1 and eTable 1 in Supplement. The average follow-up time was 14 and 12 years in the ELSA and HRS, respectively. In the SCORE2 scoring model, 2245 and 1251 participants were included from the ELSA and HRS, of whom 703 and 668 developed multimorbidity. The median SCORE2 scores were 4.55 (3.19, 6.39) and 7.18 (4.30, 10.64), respectively. The median age was 59 (56, 64) and 60 (55, 65), and the median BMI was 27.27 (24.66, 30.20) and 27.26 (24.39, 30.88). In the LE ‘8 scoring model, 2731 and 1156 participants were included from the ELSA and HRS, respectively, of whom 990 and 691 developed multimorbidity. The median LE ‘8 score was 53.33 (43.33, 61.67) and 65.83 (55.00, 75.83), and the median age was 62.00 (57.00, 69.00) and 63.00 (56.00, 70.00), respectively. In the HLS scoring model, we included 3299 and 1645 participants from the ELSA and HRS, of whom 1307 and 998 participants developed multimorbidity. The median HLS score was 3 (2, 4) and 2 (1, 3), respectively, and the median age was 62 (57, 69) and 63 (56, 70).

**Table 1:** Description of demographic characteristics and baseline health behavior scores in the ELSA and HRS

	SCORE2 ^a^			LE’8 ^b^			HLS ^b^	
	ELSA	HRS		ELSA	HRS		ELSA	HRS
	Median (*P*_25_, *P*_75_) or *N* (%)	Median (*P*_25_, *P*_75_) or *N* (%)		Median (*P*_25_, *P*_75_) or *N* (%)	Median (*P*_25_, *P*_75_) or *N* (%)		Median (*P*_25_, *P*_75_) or *N* (%)	Median (*P*_25_, *P*_75_) or *N* (%)
Case/N	703/2245	668/1251		990/2731	691/1156		1307/3299	998/1645
Total score	4.55 (3.19, 6.39)	7.18 (4.30, 10.64)		53.33 (43.33, 61.67)	65.83 (55.00, 75.83)		3.00 (2.00, 4.00)	2.00 (1.00, 3.00)
Age	59 (56, 64)	60 (55, 65)		62 (57, 69)	63 (56, 70)		62 (57, 69)	63 (56, 70)
Sex								
Male	994 (44.2)	581 (46.4)		1185 (43.4)	544 (47.1)		1423 (43.1)	776 (47.2)
Female	1251 (55.7)	760 (53.6)		1546 (56.6)	612 (52.9)		1876 (56.9)	869 (52.8)
Race								
White	2205 (98.2)	1025 (81.9)		2690 (98.5)	973 (84.2)		3254 (98.6)	1363 (82.9)
No-white	39 (1.7)	164 (13.1)		40 (1.5)	132 (11.4)		44 (1.3)	212 (12.9)
Missing	1 (0.1)	64 (5.1)		1 (<0.1)	51 (4.4)		1 (<0.1)	70 (4.3)
Drinking ^c^								
No	**-**	412 (32.9)		**-**	400 (34.6)		**-**	**-**
Yes	**-**	839 (67.1)		**-**	756 (65.4)		**-**	**-**
Last year’s alcohol intake ^c^								
No	128 (5.7)	**-**		187 (6.8)	**-**		**-**	**-**
≥1 drink/year	269 (12.0)	**-**		346 (12.7)	**-**		**-**	**-**
≥1 drink/month	247 (11.0)	**-**		310 (11.3)	**-**		**-**	**-**
≥1 drink/week	1066 (47.5)	**-**		1228 (45.0)	**-**		**-**	**-**
Almost every day	412 (18.3)	**-**		491 (18.0)	**-**		**-**	**-**
Missing	123 (5.5)	**-**		169 (6.2)	**-**		**-**	**-**
BMI	27.27 (24.66, 30.20)	27.26 (24.39, 30.88)		**-**	**-**		**-**	**-**

Abbreviations: SCORE2, Systemic coronary risk estimation 2; LE’8, Life’s essential 8; HLS, healthy life scores; BMI, Body Mass Index; ELSA, the English Longitudinal Study of Ageing; HRS, the Health and Retirement Study

^a^ The higher the score of SCORE2, the more dangerous it was

^b^ The LE’8 and HLS were reversed, with higher scores showing healthier

^c^ In ELSA, the question about drinking was set to “last year’s alcohol intake”, which has the option “no”, “more than once a year”, “more than once a month”, “more than once a week”, “almost every day”, “Missing”. In HRS, it was set to “alcohol consumption or not”

### The three lifestyle scores and multimorbidity

The associations between three lifestyle scores and multimorbidity were shown in Table 2. After adjusting for covariates, the results were robust in two cohorts. In the SCORE2, the hazard ratio (HR) of multimorbidity in the ELSA and HRS was 1.208 [95% confidence interval (CI) (1.176,1.241)] and 1.085 [95% CI (1.070, 1.101)], respectively, for each one-point increase. On the other hand, high scores were correlated with a higher risk of multimorbidity, compared to the low scores [HR = 4.125, 95% CI (3.042, 5.594) in the ELSA and HR = 2.902, 95% CI (2.353, 3.579) in the HRS]. The *E*-values of the above models were 1.401–7.715 and 1.160–5.251. In the LE ‘8, every one-point increase in scores were correlated with a lower risk of multimorbidity [HR = 0.918, 95% CI (0.882, 0.956) in the ELSA and HR = 0.981, 95% CI (0.976, 0.986) in the HRS]. Using low scores as reference, high scores were linked with lower risk of multimorbidity [HR = 0.517, 95% CI (0.303, 0.880) in the ELSA and HR = 0.432, 95% CI (0.327, 0.571) in the HRS]. The *E*-values of the above models were 1.401–7.715 and 1.160–5.251. In HLS, every increase of one point was related to the reduction of multimorbidity risk. [HR = 0.971, 95% CI (0.966, 0.976) in the ELSA and HR = 0.897, 95% CI (0.847, 0.950) in the HRS]. The risk of multimorbidity in people with high scores was related to the decrease, taking the low score as a reference. [HR = 0.369, 95% CI (0.299, 0.456) in the ELSA and HR = 0.792, 95% CI (0.633, 0.991) in the HRS]. The *E*-values of the above models were 1.401–7.715 and 1.160–5.251. The FDR of the above models were all < 0.05. We also analyzed the crude models and subgroups of the scoring models, and the results were mostly consistent (eTable 2–3 in Supplement).

**Table 2:** Associations of lifestyle scores with multimorbidity in the ELSA and HRS

Scoring models		ELSA					HRS			
		*HR* (95% *CI*)	*P* value	FDR	*E*-value		*HR* (95% *CI*)	*P* value	FDR	*E*-value
SCORE2 ^a^	Per one-point increment	1.208 (1.176, 1.241)	< 0.001	< 0.001	1.709		1.085 (1.070, 1.101)	< 0.001	< 0.001	1.389
	Low	Reference					Reference			
	Medium	2.113 (1.809, 2.468)	< 0.001	< 0.001	3.647		1.754 (1.426, 2.157)	< 0.001	< 0.001	2.904
	High	4.125 (3.042, 5.594)	< 0.001	< 0.001	7.715		2.902 (2.353, 3.579)	< 0.001	< 0.001	5.251
LE’8 ^b^	Per one-point increment	0.918 (0.882, 0.956)	< 0.001	< 0.001	1.401		0.981 (0.976, 0.986)	< 0.001	< 0.001	1.160
	Low	Reference					Reference			
	Medium	0.583 (0.513, 0.662)	< 0.001	< 0.001	2.823		0.721 (0.591, 0.879)	0.001	0.001	2.120
	High	0.517 (0.303, 0.880)	0.015	0.016	3.278		0.432 (0.327, 0.571)	< 0.001	< 0.001	4.059
HLS ^b^	Per one-point increment	0.971 (0.966, 0.976)	< 0.001	< 0.001	1.205		0.897 (0.847, 0.950)	< 0.001	< 0.001	1.473
	Low	Reference					Reference			
	Medium	0.578 (0.479, 0.697)	< 0.001	< 0.001	2.854		0.816 (0.709, 0.938)	0.004	0.005	1.751
	High	0.369 (0.299, 0.456)	< 0.001	< 0.001	4.863		0.792 (0.633, 0.991)	0.042	0.042	1.838

Abbreviations: SCORE2, Systemic coronary risk estimation 2; LE’8, Life’s essential 8; HLS, healthy life scores; FDR, False discovery rate; ELSA, the English Longitudinal Study of Ageing; HRS, the Health and Retirement Study, HR, hazard ratio; CI, confidence interval

Effect estimates were hazard ratio and 95%-confidence interval derived from the Cox regression model, which estimated the associations between different lifestyle scores and multimorbidity

^a^ SCORE2 has been classically categorized in low (1–4%), medium (5–9%), high (> 10%) risk categories. The higher the score of SCORE2, the more dangerous it was. ^b^ LE’8 has been classically categorized in low (0–49), medium (50–79), high (> 79) categories. HLS has been classically categorized in low (0–1), medium (2–3), high (4–5) categories. The LE’8 and HLS were reversed, with higher scores showing healthier

SCORE2 model was adjusted for drinking, race, and BMI; LE’8 model was adjusted for age, sex, drinking, and race; HLS model was adjusted for age, sex, and race

### Marginal predictions of three lifestyle scores and multimorbidity

**Fig. 1:**
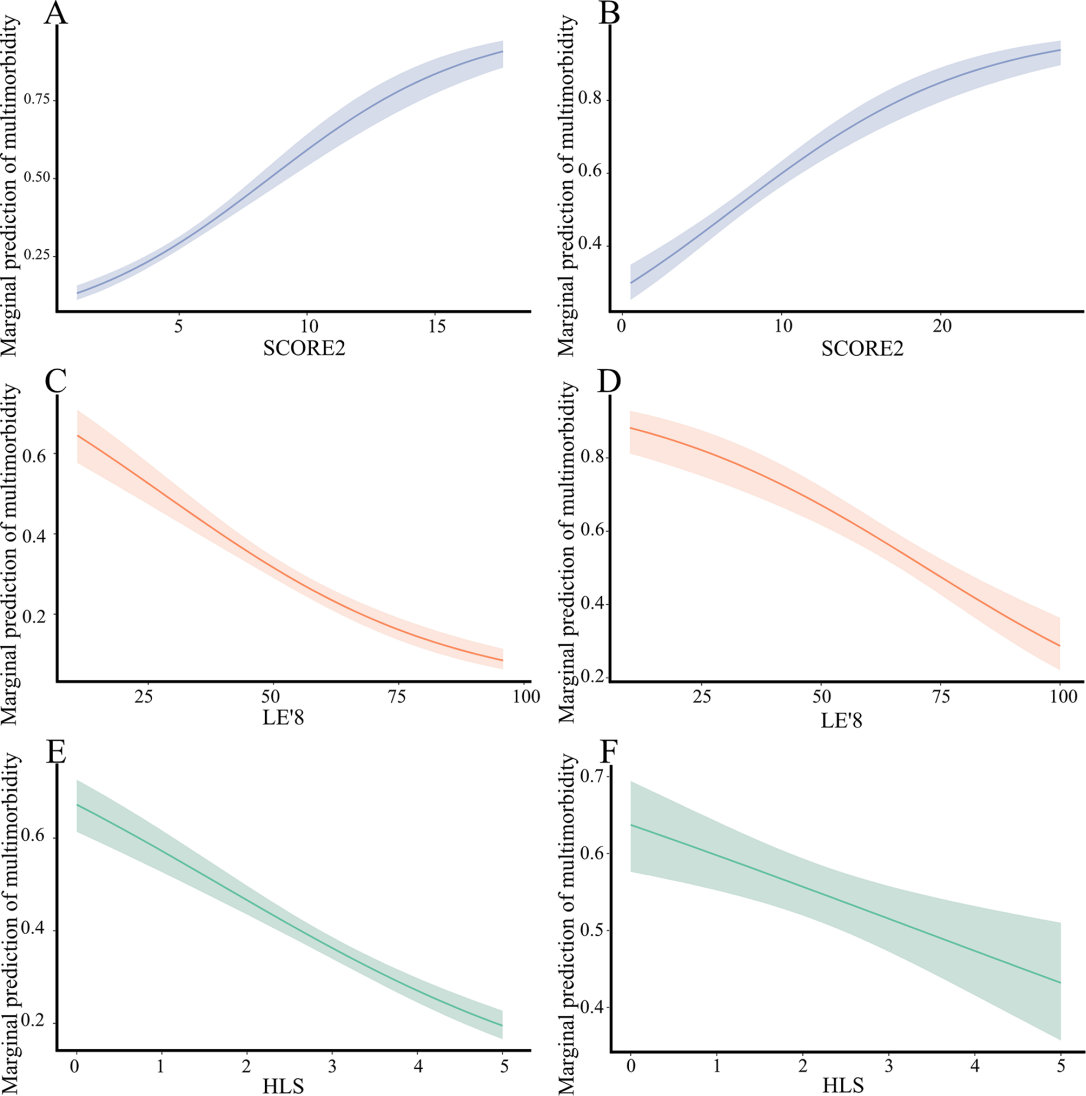
Marginal prediction between different lifestyle scores and multimorbidity in the ELSA and HRS. **A, C, E** in the English Longitudinal Study of Ageing (ELSA), **B, D, F** in the Health and Retirement Study (HRS). The higher the score of SCORE2, the more dangerous it was. The LE’8 and HLS were reversed, with higher scores showing healthier. SCORE2 model was adjusted for drinking, race, and BMI; LE’8 model was adjusted for age, sex, drinking, and race; HLS model was adjusted for age, sex, and race. Abbreviations: SCORE2, Systemic coronary risk estimation 2; LE’8, Life’s essential 8; HLS, healthy life scores; ELSA, English Longitudinal Study of Ageing; HRS, Health and Retirement Study

Marginal predictions of three lifestyle scores and multimorbidity were shown in Fig. 1. When other variables were controlled at the mean, in the SCORE2, the predicted risk of multiple disease increased for every point increase, with a coefficient of 0.052 and 0.033 in the ELSA and HRS (eTable 4 in Supplement). In the LE ‘8 and HLS, each one-point increase was negatively associated with the marginal prediction of multimorbidity.

### The trajectories of different lifestyles changes and multimorbidity

Besides, we have determined different trajectories for three lifestyle changes (eFigure 2 in Supplement), and the trajectory associations between three lifestyle changes and multimorbidity were shown in Table 3. In the SCORE2, using the low pattern as reference, the risk of multimorbidity was increased in both the moderate and crest patterns in the ELSA, which was also found in the HRS. In the LE ‘8, the risk of multimorbidity in the slow high/ high pattern was correlated with the reduced risk of multimorbidity, compared to the slow low /low pattern. The HR was 0.410 in the ELSA cohort, with 95% CI (0.307, 0.548). The HR was 0.570 in the HRS, with 95% CI (0.438, 0.743). In the HLS, the HR of multimorbidity in the slow high/high model, compared to the low pattern, was 0.868 in the ELSA, with 95% CI (0.639, 1.180), and 0.734 in the HRS, with 95%CI (0.593, 0.908).

**Table 3:** The trajectory results of different lifestyle changes and risk of multimorbidity in ELSA and HRS

Scoring models		ELSA					HRS			
		Crude	*P* value	Adjusted	*P* value		Crude	*P* value	Adjusted	*P* value
SCORE2	Low pattern	Reference		Reference		Low pattern	Reference		Reference	
	Moderate pattern	2.299 (1.451, 3.643)	< 0.001	2.172 (1.370, 3.445)	< 0.001	Moderate pattern	1.623 (1.258, 2.093)	< 0.001	1.598 (1.236, 2.067)	< 0.001
	Trough pattern	3.971 (2.166, 7.279)	< 0.001	4.024 (2.192, 7.387)	< 0.001	Crest pattern	2.991 (2.071, 4.320)	< 0.001	2.905 (2.003, 4.213)	< 0.001
	Crest pattern	5.185 (2.994, 8.978)	< 0.001	5.602 (3.227, 9.724)	< 0.001					
LE’8	Slow low pattern	Reference		Reference		Low pattern	Reference		Reference	
	Slow high pattern	0.442 (0.333, 0.587)	< 0.001	0.410 (0.307, 0.548)	< 0.001	High pattern	0.589 (0.454, 0.766)	< 0.001	0.570 (0.438, 0.743)	< 0.001
HLS	Slow low pattern	Reference		Reference		Slow low pattern	Reference		Reference	
	High pattern	0.847 (0.624, 1.150)	0.287	0.868 (0.639, 1.180)	0.367	High pattern	0.821 (0.665, 1.012)	0.065	0.734 (0.593, 0.908)	0.004

Abbreviations: SCORE2, Systemic coronary risk estimation 2; LE’8, Life’s essential 8; HLS, healthy life scores; ELSA, the English Longitudinal Study of Ageing; HRS, the Health and Retirement Study

Effect estimates were hazard ratio and 95%-confidence interval derived from Cox regression model, which estimated the associations between different lifestyle scores and multimorbidity

The latent class trajectory model (LCTM) was used to identify the trajectory of the three lifestyle changes over time. We identified five healthy lifestyle trajectories in these two cohorts: slow low/low, moderate, slow high/high, crest, and trough pattern. The trough pattern was defined as U-shape. The crest pattern was reversed, as an inverted U-shape

The higher the score of SCORE2, the more dangerous it was. The LE’8 and HLS were reversed, with higher scores showing healthier

SCORE2 model was adjusted for drinking, race, and BMI; LE’8 model was adjusted for age, sex, drinking, and race; HLS model was adjusted for age, sex, and race

### The RMST of three lifestyle scores and multimorbidity

**Fig. 2:**
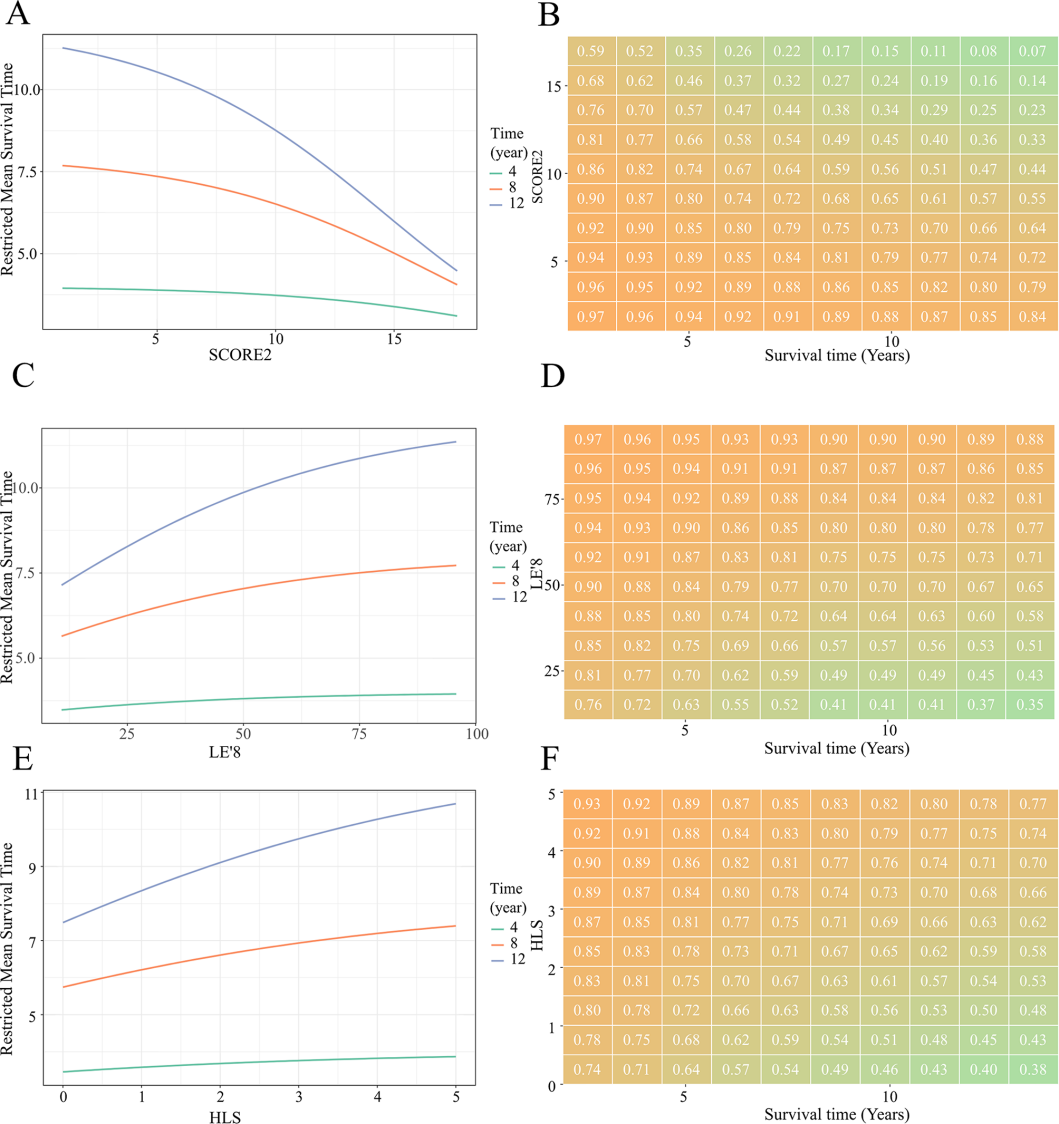
The restricted mean survival time between different lifestyle scores and multimorbidity in the ELSA. **A, C, E** were the restriction mean survival time of the scoring models for SCORE2, LE’8, and HLS, respectively. **B, D, F** were the survival time of the scoring models for SCORE2, LE’8, and HLS, respectively. The green, orange, purple line represented the results of a 4, 8, 12-year follow-up, respectively. The higher the score of SCORE2, the more dangerous it was. The LE’8 and HLS were reversed, with higher scores showing healthier. SCORE2 model was adjusted for drinking, race, and BMI; LE’8 model was adjusted for age, sex, drinking, and race; HLS model was adjusted for age, sex, and race. Abbreviations: SCORE2, Systemic coronary risk estimation 2; LE’8, Life’s essential 8; HLS, healthy life scores; ELSA, English Longitudinal Study of Ageing; HRS, Health and Retirement Study

On the other hand, some Cox regression models did not comply with the proportional hazards assumption in ELSA. We analyzed the RMST and survival matrix, and the results were shown in Fig. 2. At 4, 8, and 12 years of follow-up, the mean survival time was not proportional (Fig. 2A, C, and E). In the SCORE2, the probability of survival gradually decreased with longer follow-up, and the reduction in survival probability was much greater in the exposed group with a score of > 10 than in the group with a score of 5 (Fig. 2B). In LE ‘8 and HLS, the probability of survival gradually decreased with longer follow-up, and the probability of survival decreased in exposed groups with higher scores slowly (Fig. 2D and F). It shows that adhering to a long-term healthy lifestyle can improve the survival probability of the population.

### Mediation analysis

**Fig. 3:**
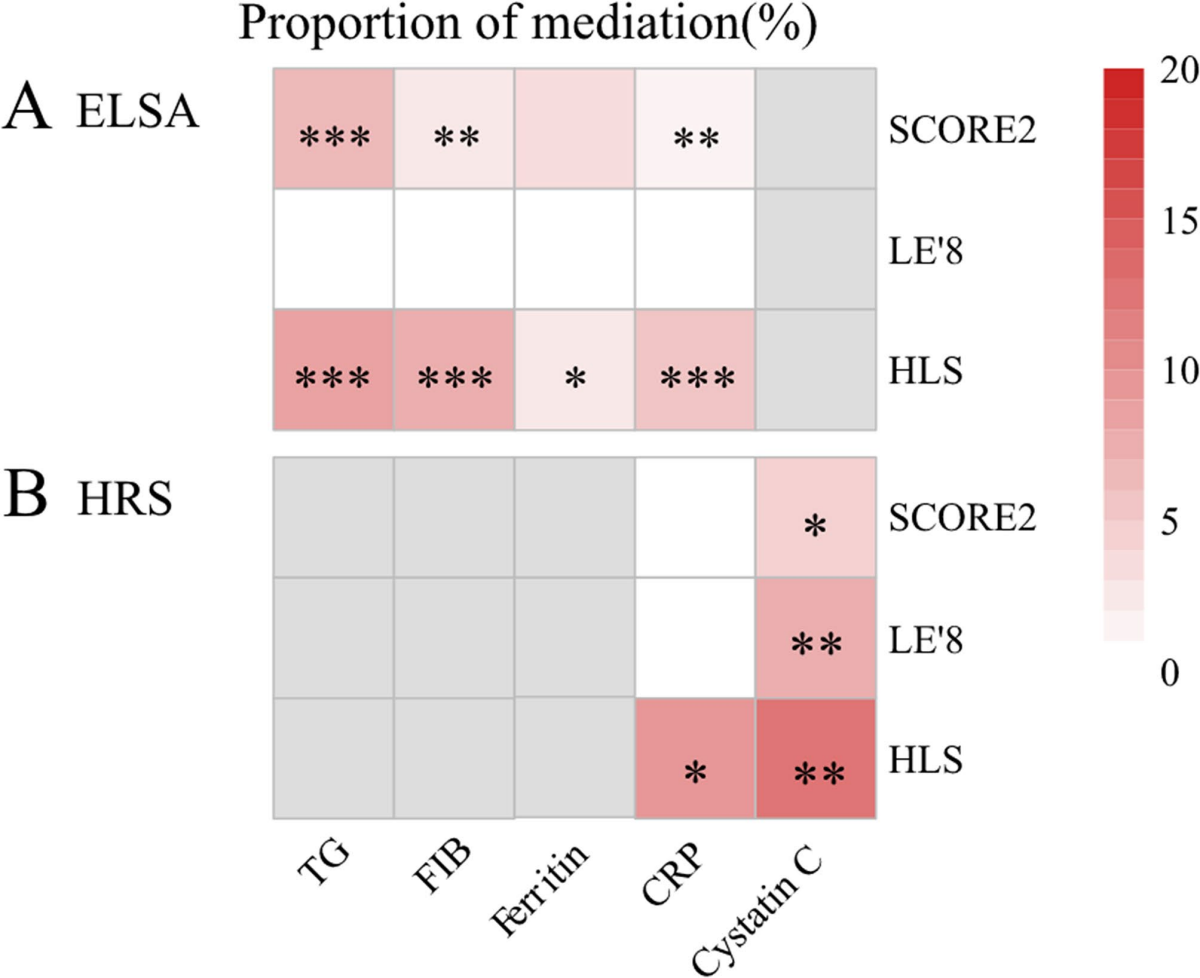
Association between different lifestyle scores, mediation variables, and risk of multimorbidity in the ELSA and HRS. (**A**) in the English Longitudinal Study of Ageing (ELSA), (**B**) in the Health and Retirement Study (HRS). SCORE2 model was adjusted for drinking, race, and BMI; LE’8 model was adjusted for age, sex, drinking, and race; HLS model was adjusted for age, sex, and race. All of these were used logistic regression models. The depth of the square color indicated the proportion of intermediaries. The grey squares indicated that no data is available. “*” for *P* <0.05; “**” for *P* <0.01; “***” for *P* <0.001. Abbreviations: SCORE2, Systemic coronary risk estimation 2; LE’8, Life’s essential 8; HLS, healthy life scores; TG, triglyceride; CRP, C-reaction protein, FIB, fibrinogen; ELSA, English Longitudinal Study of Ageing; HRS, Health and Retirement Study

The mediating analysis highlighted that triglyceride (TG), C-reaction protein (CRP), fibrinogen, and Cystatin C may partly explain the correlation between healthy lifestyle and multimorbidity (mediating percentages ranged from 0.06% to 12,76%, Fig. 3) in the ELSA and HRS.

### Sensitivity analysis

First, we perform intercept correction on the SCORE2 model. By adjusting the intercept of the model, the calibrated predicted mean was close to the actual observed mean, and the calibration degree of the model is improved. The results are consistent with the main results (eTable 5 and eFigure 3 in Supplement). Second, the results were robust by removing baseline disease and those who developed multimorbidity within two years (eTable 6 in Supplement). Third, the population is re-analyzed by inverse probability weighting, and the result remains unchanged (eTable 7 in Supplement). Fourthly, the competitive risk model with mortality is used for analysis, and the results are robust (eTable 8 in Supplement).

## Discussion

To our knowledge, this was the first study to investigate the link between multiple healthy lifestyle patterns and their change trajectories and multimorbidity in the same context. Based on two large national cohort studies, ELSA and HRS, we found that participants with higher SCORE2 scores a had heightened risk of multimorbidity. While higher scores in LE ‘8 and HLS were accompanied by a decreased of multimorbidity. Moreover, in the LCMT model, with low pattern as reference, moderate, trough and crest patterns were related to an elevated risk of multimorbidity in the SCORE2, and slow high/high pattern may decrease the risk of multimorbidity in the LE ‘8 and HLS. These results suggested that adhering to a healthy life can reduce the risk of disease, and the longer the adherence, the better the quality of life. The results of mediation analysis suggested that TG, CRP, fibrinogen, and Cystatin C may partially mediate the link between healthy lifestyle and multimorbidity, providing clues for the study mechanism.

We found that an increase in SCORE2 resulted in an elevated risk of multimorbidity. Multiple Mendelian studies have found that higher genetically predicted SBP levels were positively associated with the risk of major CVD types [20, 21]. Triglyceride-rich lipoprotein cholesterol levels may affect the development of atherosclerosis, which poses a health threat [22]. At the same time, previous study has shown that SCORE2 is also suitable for predicting all-cause dementia, Alzheimer’s disease, and all-cause mortality in European populations ^18^. Our study confirmed that it was also feasible in predicting multimorbidity, expanding the scope of research evidence. In addition, we applied the SCORE2 to the HRS based on previous study [19] to expand the diversity of the population. meanwhile, we corrected the intercept of SCORE2 based on the queue data, and the results were still robust. However, the algorithm considered health indicators and did not specifically reflect the extent to which lifestyle behaviors affected disease. Therefore, the present study considered the association between LE ‘8 and multimorbidity, which included health behaviors and indicators, and each indicator has detailed measurement and quantitative criteria.

We found that increased LE’ 8 and HLS scores lowered the likelihood of multimorbidity. Previous studies have found that an ideal CVH (higher LE ‘8) lower both the relative risk of CVD and all-cause mortality [22–24], and was significantly linked to a decreased risk of new non-alcoholic fatty liver disease [26]. Moreover, the marginal prediction results of this study also showed that LE ‘8 and HLS scoring models were negatively correlated with the predicted risk of multimorbidity, indicating the robustness of the results of this study. Besides, health behaviors did not exist independently of each other. Among them, PA connected with a decreasing risk of mortality from CVD [27], and PA intensity may have a greater impact on reducing CVD risk than the total PA volume [28]. In addition, obesity was associated with various metabolic disorders and physical enlargement of various tissues and organs, and preventable mortality worldwide can be attributed to obesity and sedentary lifestyles [29]. Two or more healthy lifestyle can compensate for the harmful effects on lipid metabolites [30]. Low PA exacerbated the harmful association of sleep deprivation with the risk of all-cause and cause-specific mortality [31]. However, we identified some subgroup outcomes as incongruent with prior studies, implying potential intergroup heterogeneity or instability stemming from inadequate statistical power. Consequently, it is imperative to enlarge the sample size and further validate the results in specific population subgroups.

Through the different trajectory fitting of three lifestyle changes, we found that long-term adherence to healthier behaviors was conducive to reducing disease risk and maintaining a good quality of life. A UK Biobank study found that when people improved their lifestyle, their risk of developing ischemic heart diseases decreased [32]. A large prospective cohort study also found that maintaining a high level of PA or improving PA could help prevent mortality from cancer and other causes [33]. This was consistent with our findings. We found that improvement of unhealthy lifestyle (crest pattern) and deterioration of a healthy lifestyle (trough pattern) were associated with different risks of multimorbidity compared to a healthy lifestyle, and deterioration of healthy lifestyle (trough pattern) had a greater impact on multimorbidity risk. The result of RMST also supported the above findings. From a policy perspective, the findings underscore the significance of adopting healthy lifestyles to prevent multiple health issues in the long term. It is crucial to acknowledge that health benefits are not immediate and require consistent effort and commitment, particularly for individuals who have previously struggled with sustaining a healthy routine but eventually abandoned their efforts.

Finally, we found that TG, CRP, fibrinogen, and Cystatin C may partially mediate the association between healthy lifestyle and multimorbidity. CRP was the main protein in acute phase reaction and a minimally invasive indicator of inflammatory response [34]. The study found that lifestyle risk factors can influence CRP through the nuclear factor kappa-B signaling pathway, which can adversely affect chronic diseases (diabetes, cancer, and CVD, among others) [35]. Obesity, excessive alcohol consumption and prolonged inactivity can cause elevated triglyceride levels and accompanying lipoprotein changes, which can affect atherosclerotic cardiovascular disease [36]. This is consistent with our findings. However, there is a need for further research to explore the underlying mechanisms.

The advantages of this study were as follows. First, this was the first study to investigate the link between different healthy lifestyle models and multimorbidity based on repeated measurements, providing evidence for reducing disease risk and health care burden through lifestyle changes. Second, the study was based on two nationally representative cohorts in the United States and the United Kingdom, with long-term follow-up. Third, we applied the new SCORE2, LE’8, and HLS scoring models as useful tools for evaluating multimorbidity. Fourth, our results were robust, with broadly consistent findings across two independent cohorts. Fifth, we also evaluated potentially mediating elements to contribute evidence for future mechanistic studies.

However, there were some limitations to our study. First, the use of self-reported behavior patterns rather than direct measurement by objective equipment. Follow-up studies could objectively evaluate participants’ lifestyle behaviors by wearing electronic devices. Second, most of the participants in this study were of white race, so further research on other races is needed to ensure the results are implementable Third, the study excluded a considerable number of subjects during the analysis, which could lead to potential selection bias. However, our results were consistent across the two cohort studies. In addition, we still found robust results in sensitivity analyses (inverse probability weighting and competing risk models). Fourth, because this study was observational, causation cannot be well explained. The follow-up could be studied in depth using randomized controlled trials. Finally, although we calculate the *E*-value, we cannot rule out the effect of unmeasured or residual confounding.

This study explored the impact of a healthy lifestyle on multimorbidity in response to the severe challenges of aging. In the future, it is necessary to conduct in-depth research through intervention measures, broaden the research population and race, and study health behaviors throughout the entire life cycle. Lifestyle is one of the modifiable factors, clarify the two relationships, and may strive to achieve greater health equity.

## Conclusions

Overall, we found that three healthy lifestyle scores (SCORE2, LE’8, HLS) were associated with multimorbidity. In the LCMT model, changes in healthy lifestyle were associated with different risks of multimorbidity, and long-term adherence to a healthy lifestyle was conducive to maintaining a good quality of life. In addition, TG, CRP, fibrinogen, and Cystatin C may partially mediate the link between healthy lifestyle and multimorbidity, providing clues to the mechanism.

## Electronic supplementary material

Below is the link to the electronic supplementary material.

Supplementary Material 1

Supplementary Material 2

## Data Availability

All processed data generated or used during the study appear in the submitted article or supplementary material. The ELSA, and HRS data can be requested via the study websites (https://www.elsa-project.ac.uk/; https://hrs.isr.umich.edu/).
