# Supplementary material for: Association between healthy lifestyle on life course and multimorbidity in adults: results from two national prospective cohort studies
Source: BMC Public Health. 2024 Oct 23;24:2942. doi: 10.1186/s12889-024-20443-7 (PMC11515530; doi:10.1186/s12889-024-20443-7)
Supplement: Supplementary file 2 — Supplementary Material 2 [file 12889_2024_20443_MOESM2_ESM.docx]

**Supplementary Material**

**Association between healthy lifestyle on life course and multimorbidity in adults:** **results from two national prospective cohort studies**

**Supplementary Methods**

**eTable 1.** Associations of different lifestyle scores components (LE’8 and HLS) with multimorbidity in two cohorts.

**eTable 2.** Associations of different lifestyle scores components (LE’8 and HLS) with multimorbidity in two cohorts.

**eTable 3.** Associations of different lifestyle scores with multimorbidity in two cohorts.

**eTable 4.** Simple Slopes between different lifestyle scores and multimorbidity in two cohorts.

**eTable 5.** The differentiation and calibration of SCORE2 prediction model in updated datasets in two cohorts.

**eTable 6.** Associations of different lifestyle scores with multimorbidity (＞2 years) in two cohorts.

**eTable 7.** Associations of different lifestyle scores with multimorbidity by using Inverse probability weighting in two cohorts.

**eTable 8.** Comparison of the risk of different lifestyle scores with multimorbidity in the original Cox model vs. the competing-risks regression model.

**eFigure 1.** Flowchart of the participants selection.

**eFigure 2.** The trajectory grouping results of different lifestyle scores and risk of multimorbidity in two cohorts.

**eFigure 3.** The differentiation and calibration of SCORE2 prediction model in updated datasets in two cohorts.

**Supplementary Methods**

The SCORE2 was a new algorithm derived, calibrated, and validated to predict the 10-year risk of first-onset CVD in a European population aged 40-69 years with no prior history of cardiovascular disease. SCORE2 is an optimization based on SCORE. SCORE only includes fatal cardiovascular disease outcomes, which means it underestimates the total cardiovascular disease burden and may misestimate the risk. SCORE2 can appropriately avoid the above limitations. The risk calculation for a European man or woman without diabetes was shown in Supplementary methods Table 1.

Life’s essential 8 is a re-rating of the original 7 cardiovascular health (CVH) indicators and the new sleep indicators on a more continuous scale to better explain inter individual differences and intra individual changes (Supplementary methods Table 2). The writing group suggests that a total score of 80 to 100 for CVH is considered high; 50~79, moderate CVH; 0-49 points, low CVH.

The flowchart of the statistical analysis was showed in Supplementary methods eFigure 1.

**Supplementary methods eTable 1: Illustration of SCORE2 risk estimation for a non-diabetic man or woman with given risk factor values.**

| 1.Calculation of Linear Predictor | | | | | |
| --- | --- | --- | --- | --- | --- |
| Risk factor (units) | Risk Factor Value | Transformed value | Log HRx transformed value | | |
|  |  |  | Male | | Female |
| Age (years) | 50 | (50-60)/5=-2 | 0.3742×-2=-0.7484 | | 0.4648×-2=-0.9296 |
| Smoking (yes or no) | yes | 1 | 0.6012 × 1 = 0.6012 | | 0.7744 × 1 = 0.7744 |
| SBP (mm Hg) | 140 | (140 - 120)/20 = 1 | 0.2777 × 1 = 0.2777 | | 0.3131 × 1 = 0.3131 |
| Total cholesterol (mmol/L) | 6.3 | (6.3 - 6)/1 = 0.3 | 0.1458 × 0.3 = 0.04374 | | 0.1002 × 0.3 = 0.03006 |
| HDL (mmol/L) | 1.4 | (1.4- 1.3)/0.5 = 0.2 | -0.2698 × 0.2 = -0.05396 | | -0.2606 × 0.2 = -0.05212 |
| Smoking × age interaction |  | -2 × 1 = -2 | -0.0755 × -2 = 0.151 | | -0.1088 × -2 = 0.2176 |
| SBP × age interaction |  | -2 × 1 = -2 | -0.0255 × -2 = 0.051 | | -0.0277 × -2 = 0.0554 |
| Total cholesterol × age interaction | | -2 × 0.3 = -0.6 | -0.0281 × -0.6 = 0.01686 | | -0.0226 × -0.6 = 0.01356 |
| HDL cholesterol × age interaction | | -2 × 0.2 = -0.4 | 0.0426 × -0.4 = -0.01704 | | 0.0613 × -0.4 = -0.02452 |
| Linear predictor: | | | ∑ = 0.3221 | | ∑ = 0.39788 |
| 2) 10-year risk estimation (un-calibrated) = 1-basesurv ^exp (linear predictor)^ | | | 1-0.9605^exp (0.3221)^ = 0.0541 | | 1-0.9776^exp (0.39788)^ = 0.0332 |
| 3) Calibration of risk estimate according to region specific scaling factors Calibrated 10-year risk = 1-exp (-exp (scale1 + scale2 × ln (-ln (1-un-calibrated 10-year risk)) | | | | | |
| Risk region | | Male | | Female | |
| Low |  | 1-exp (-exp (-0.5699+0.7476 × ln (-ln (1-0.0541)))) = 0.0631 | | 1-exp (-exp (-0.7380+0.7019 × ln (-ln (1-0.0332)))) = 0.0434 | |
| Moderate |  | 1-exp (-exp (-0.1565+0.8009 × ln (-ln (1-0.0541)))) = 0.0811 | | 1-exp (-exp (-0.3143+0.7701 × ln (-ln (1-0.0332)))) = 0.0523 | |
| High |  | 1-exp (-exp (0.3207+0.9360 × ln (-ln (1-0.0541)))) = 0.0881 | | 1-exp (-exp (0.5710+0.9369 × ln (-ln (1-0.0332)))) = 0.0713 | |
| Very high |  | 1-exp (-exp (0.5836+0.8294 × ln (-ln (1-0.0541)))) = 0.1506 | | 1-exp (-exp (0.9412+0.8329 × ln (-ln (1-0.0332)))) = 0.1414 | |

Abbreviations: SCORE2, Systemic coronary risk estimation 2; SBP, Systolic blood pressure; HDL, high-density lipoprotein.

**Supplementary methods eTable 2: The quantification score of CVH (LE’8) metric and the quantification score of this study.**

| Domain | CVH metric | Quantification of CVH metric: adults (≥20 y of age) | Quantification of CVH metric in this study |
| --- | --- | --- | --- |
| Health behaviors | PA | Metric: Minutes of moderate- (or greater)  intensity activity per week  Scoring:  Points Minutes  100 ≥150  90 120–149  80 90–119  60 60–89  40 30–59  20 1–29  0 0 | Metric: Frequency of moderate- (or greater)  Points Frequency  100 Vigorous and moderate sports more than once a week  90 Vigorous sports more than once a week or vigorous sports 1-4 times a month  80 Vigorous sports more than once a week or vigorous sports once a week and moderate sports more than once a week  60 Vigorous and moderate sports once a week or vigorous sports 1-3 times a month and moderate sports more than once a week  40 Vigorous sports once a week or vigorous sports 1-3 times a month and moderate sports once a week  20 Moderate sports once a week  0 hardly or never |
|  | Nicotine exposure | Metric: Combustible tobacco use or inhaled  NDS use; or secondhand smoke exposure  Scoring:  Points Status  100 Never smoker  75 Former smoker, quit ≥5 y  50 Former smoker, quit 1–<5 y  25 Former smoker, quit <1 y, or  currently using inhaled NDS  0 Current smoker  Subtract 20 points (unless score is 0) for living with active indoor smoker in home | Metric: Combustible tobacco use or inhaled  Scoring:  Points Status  100 Never smoker  75 Former smoker, quit ≥5 y  50 Former smoker, quit 1–<5 y  25 Former smoker, quit <1 y 0 Current smoker |
|  | Sleep health | Metric: Average hours of sleep per night  Scoring:  Points Level  100 7–<9  90 9–<10  70 6–<7  40 5–<6 or ≥10  20 4–<5  0 <4 | Missing this indicator |
|  | Diet | Quantiles of DASH-style diet adherence  or HEI-2015 (population)  Scoring (population):  Points Quantile  100 ≥95th percentile (top/ideal diet)  80 75th–94th percentile  50 50th–74th percentile  25 25th–49th percentile  0 1st–24th percentile (bottom/  least ideal quartile)  Scoring (individual):  Points MEPA score (points)  100 15–16  80 12–14  50 8–11  25 4–7  0 0–3 | Missing this indicator |
| Health factors | BMI | Metric: BMI (kg/m^2^)  Scoring:  Points Level  100 <25  70 25.0–29.9  30 30.0–34.9  15 35.0–39.9  0 ≥40.0 | Metric: BMI (kg/m^2^)  Scoring:  Points Level  100 <25  70 25.0–29.9  30 30.0–34.9  15 35.0–39.9  0 ≥40.0 |
|  | Blood lipids | Metric: Non–HDL cholesterol (mg/dL)  Scoring:  Points Level  100 <130  60 130–159  40 160–189  20 190–219  0 ≥220  If drug-treated level, subtract 20 points | Metric: Non–HDL cholesterol (mg/dL)  Scoring:  Points Level  100 <130  60 130–159  40 160–189  20 190–219   1. ≥220 |
|  | Blood glucose | Metric: FBG (mg/dL) or HbA1c (%)  Scoring:  Points Level  100 No history of diabetes and  FBG <100 (or HbA1c <5.7)  60 No diabetes and FBG  100–125 (or HbA1c 5.7–6.4)  (prediabetes)  40 Diabetes with HbA1c <7.0  30 Diabetes with HbA1c 7.0–7.9  20 Diabetes with HbA1c 8.0–8.9  10 Diabetes with Hb A1c 9.0–9.9  0 Diabetes with HbA1c ≥10.0 | Metric: HbA1c (%)  Scoring:  Points Level  100 HbA1c <5.7  60 HbA1c 5.7–6.4  40 HbA1c <7.0  30 HbA1c 7.0–7.9  20 HbA1c 8.0–8.9  10 Hb A1c 9.0–9.9  0 HbA1c ≥10.0 |
|  | Blood pressure | Metric: Systolic and diastolic BPs  (mmHg)  Scoring:  Points Level  100 <120/<80 (optimal)  75 120–129/<80 (elevated)  50 130–139 or 80–89 (stage 1  hypertension)  25 140–159 or 90–99  0 ≥160 or ≥100  Subtract 20 points if treated level | Metric: Systolic and diastolic BPs  (mmHg)  Scoring:  Points Level  100 <120/<80 (optimal)  75 120–129/<80 (elevated)  50 130–139 or 80–89  25 140–159 or 90–99  0 ≥160 or ≥100 |

Abbreviations: CVH, cardiovascular health; LE’8, Life’s essential 8; PA, physical activity; BMI, body mass index; HbA1c, glycated hemoglobin.

**
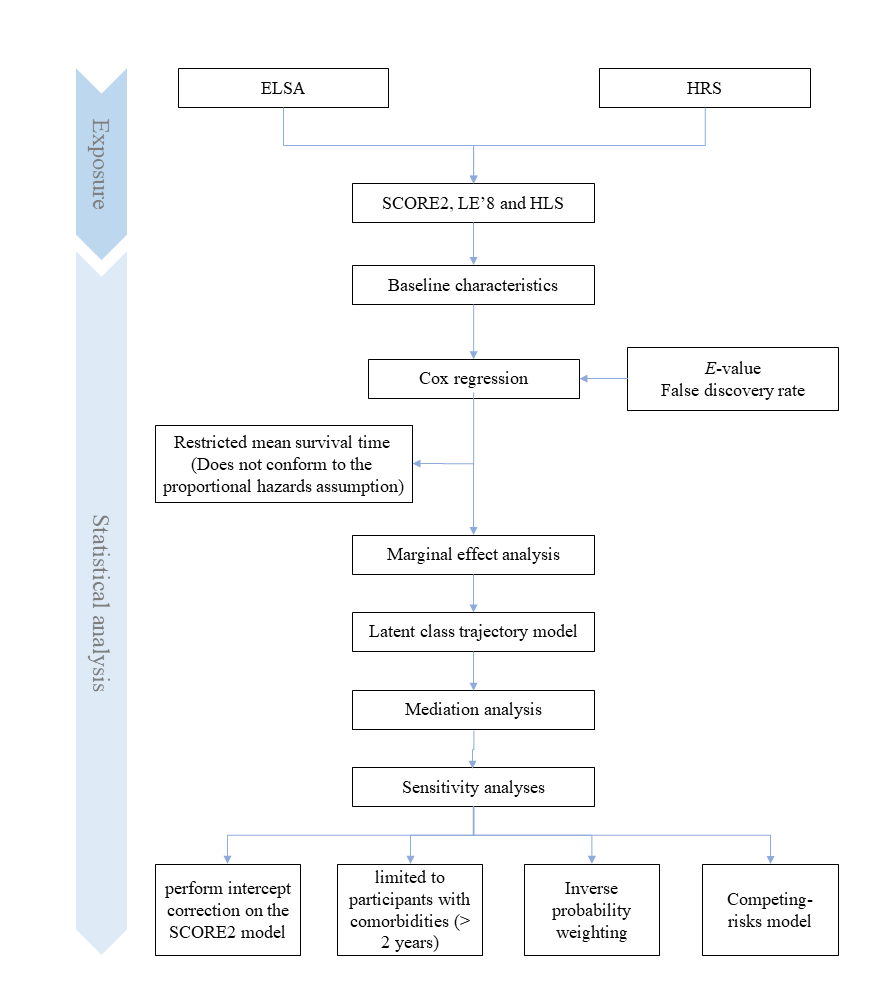
**

**Supplementary methods eFigure 1. Flowchart of the statistical analysis.** Abbreviations: SCORE2, Systemic coronary risk estimation 2; LE’8, Life’s essential 8; HLS, healthy life scores; ELSA, English Longitudinal Study of Ageing; HRS, Health and Retirement Study.

**eTable 1. Description of the components of the baseline health behavior score in the ELSA and HRS.**

| Scores | Components | ELSA | HRS |
| --- | --- | --- | --- |
|  |  | Median (*P*_25_, *P*_75_) or N (%) | Median (*P*_25_, *P*_75_) or N (%) |
| SCORE2 ^a^ | | 4.55 (3.19, 6.39) | 7.18 (4.30, 10.64) |
| LE'8 ^b^ | Smoking | 0 (0, 0) | 75 (0, 100) |
|  | PA | 40 (40, 80) | 40 (40, 80) |
|  | BMI | 70 (30, 100) | 70 (30, 100) |
|  | BP | 50 (25, 75) | 50 (25, 100) |
|  | Non-HDL | 40 (20, 60) | 60 (40, 100) |
|  | HbA1c | 100 (60, 100) | 100 (60, 100) |
| HLS ^b^ | Smoking |  |  |
|  | No | 2867 (86.91) | 451 (72.58) |
|  | Yes | 432 (13.09) | 1194 (27.42) |
|  | Drinking |  |  |
|  | No | 1136 (34.43) | 1084 (65.90) |
|  | Yes | 2163 (65.57) | 561 (34.10) |
|  | Sleeping well |  |  |
|  | No | 1377 (41.74) | 648 (39.39) |
|  | Yes | 1922 (58.26) | 997 (60.61) |
|  | PA |  |  |
|  | No | 557 (16.88) | 1003 (60.97) |
|  | Yes | 2742 (83.12) | 642 (39.03) |
|  | Obesity |  |  |
|  | No | 973 (29.49) | 435 (26.44) |
|  | Yes | 2326 (70.51) | 1210 (73.56) |

Abbreviations: SCORE2, Systemic coronary risk estimation 2; LE’8, Life’s essential 8; HLS, healthy life scores; BMI, Body Mass Index; ELSA, the English Longitudinal Study of Ageing; HRS, the Health and Retirement Study, PA, physical activity; BMI, body mass index; BP, blood pressure; Non-HDL, Non-high density lipoprotein; HbA1c, glycosylated hemoglobin.

^a^ The higher the score of SCORE2, the more dangerous it was.

^b^ The LE’8 and HLS were reversed, with higher scores showing healthier.

**eTable 2. Associations of different lifestyle scores components (LE’8 and HLS) with multimorbidity in two cohorts.**

| Scoring models | Components | ELSA | | | |  | HRS | | | |
| --- | --- | --- | --- | --- | --- | --- | --- | --- | --- | --- |
|  |  | Crude | *P* value | Adjusted | *P* value |  | Crude | *P* value | Adjusted | *P* value |
| LE'8 | Smoke | 1.005 (1.003, 1.006) | 7.88E-08 | 1.007 (1.006, 1.009) | < 2e-16 |  | 0.999 (0.997, 1.000) | 9.01E-02 | 0.997 (0.995, 0.999) | 1.66E-03 |
|  | PA | 0.988 (0.986, 0.990) | <2e-16 | 0.990 (0.988, 0.992) | < 2e-16 |  | 0.997 (0.995, 0.999) | 6.45E-03 | 0.997 (0.995, 0.999) | 1.4E-02 |
|  | BMI | 0.991 (0.989, 0.993) | <2e-16 | 0.990 (0.988, 0.992) | < 2e-16 |  | 0.997 (0.994, 0.999) | 1.29E-02 | 0.996 (0.994, 0.999) | 2.39E-03 |
|  | BP | 0.985 (0.983, 0.987) | <2e-16 | 0.988 (0.986, 0.990) | < 2e-16 |  | 0.991 (0.988, 0.993) | < 2e-16 | 0.993 (0.991, 0.996) | 4.72E-09 |
|  | Non-HDL | 1.002 (1.000, 1.004) | 3.71E-02 | 1.001 (0.999, 1.003) | 4.42E-01 |  | 1.001 (0.998, 1.003) | 6.14E-01 | 1.000 (0.997, 1.002) | 9.22E-01 |
|  | HbA1c | 0.981 (0.979, 0.984) | <2e-16 | 0.983 (0.980, 0.986) | < 2e-16 |  | 0.989 (0.986, 0.992) | 1.72E-11 | 0.991 (0.988, 0.994) | 1.33E-07 |
| HLS | Smoke | 0.657 (0.571, 0.760) | 1.21E-08 | 0.508 (0.438, 0.587) | < 2e-16 |  | 1.127 (0.983, 1.292) | 8.75E-02 | 1.446 (1.255, 1.665) | 3.14E-07 |
|  | Drink | 1.494 (1.338, 1.668) | 9.51E-13 | 1.409 (1.258, 1.578) | 3.04E-09 |  | 0.817 (0.718, 0.930) | 2.17E-03 | 0.820 (0.720, 0.933) | 2.65E-03 |
|  | Sleeping well | 0.724 (0.650, 0.807) | 5.71E-09 | 0.674 (0.604, 0.752) | 1.97E-12 |  | 0.893 (0.787, 1.013) | 7.73E-02 | 0.816 (0.718, 0.928) | 1.97E-03 |
|  | PA | 0.496 (0.437, 0.563) | <2e-16 | 0.549 (0.483, 0.624) | < 2e-16 |  | 0.784 (0.689, 0.893) | 2.34E-04 | 0.768 (0.674, 0.876) | 7.83E-05 |
|  | Obesity | 0.551 (0.482, 0.630) | <2e-16 | 0.570 (0.498, 0.654) | 7.89E-16 |  | 0.765 (0.661, 0.886) | 3.48E-04 | 0.793 (0.683, 0.921) | 2.39E-03 |

Abbreviations: LE’8, Life’s essential 8; HLS, healthy life scores; ELSA, the English Longitudinal Study of Ageing; HRS, the Health and Retirement Study; PA, physical activity; BMI, Body Mass Index; BP, blood pressure; HbA1c, Hemoglobin A1c; Non-HDL, non-high-density lipoprotein cholesterol.

Effect estimates were hazard ratio and 95%-confidence interval derived from Cox regression model, which estimated the associations between different lifestyle scores components (LE’8 and HLS) and multimorbidity.

The higher the score of LE’8 and HLS, the healthier it was. In HLS, unhealthy lifestyles were used as a reference.

The crude model adjusted nothing. LE’8 model was adjusted for age, sex, drinking, and race; HLS model was adjusted for age, sex, and race.

**eTable 3. Associations of different lifestyle scores with multimorbidity in two cohorts.**

| Scoring models | | ELSA | | | |  | HRS | | | |
| --- | --- | --- | --- | --- | --- | --- | --- | --- | --- | --- |
|  |  | Crude | *P* value | FDR | *E*-value |  | Crude | *P* value | FDR | *E*-value |
| SCORE2 ^a^ | Per one-point increment | 1.221 (1.189, 1.254) | <2e-16 | 4.50E-16 | 1.740 |  | 1.084 (1.069, 1.099) | <2e-16 | 4.50E-16 | 1.386 |
|  | Low | Reference |  |  |  |  | Reference |  |  |  |
|  | Medium | 2.200 (1.884, 2.568) | <2e-16 | 4.50E-16 | 3.825 |  | 1.791 (1.456, 2.203) | 3.37E-08 | 5.27E-08 | 2.981 |
|  | High | 4.531 (3.344, 6.141) | <2e-16 | 4.50E-16 | 8.531 |  | 2.979 (2.417, 3.672) | < 2e-16 | 4.50E-16 | 5.407 |
| LE’8 ^b^ | Per one-point increment | 0.967 (0.962, 0.972) | <2e-16 | 4.50E-16 | 1.222 |  | 0.981 (0.976, 0.986) | 9.30E-14 | 1.86E-13 | 1.160 |
|  | Low | Reference |  |  |  |  | Reference |  |  |  |
|  | Medium | 0.527 (0.465, 0.598) | < 2e-16 | 4.50E-16 | 3.203 |  | 0.779 (0.640, 0.948) | 1.28E-02 | 1.36E-02 | 1.887 |
|  | High | 0.357 (0.210, 0.607) | 0.000141 | 1.81E-04 | 5.047 |  | 0.411 (0.312, 0.542) | 2.77E-10 | 4.99E-10 | 4.300 |
| HLS ^b^ | Per one-point increment | 0.754 (0.712, 0.797) | <2e-16 | 4.50E-16 | 1.984 |  | 0.878 (0.829, 0.929) | 6.03E-06 | 8.35E-06 | 1.537 |
|  | Low | Reference |  |  |  |  | Reference |  |  |  |
|  | Medium | 0.646 (0.536, 0.778) | 4.28E-06 | 6.16E-06 | 2.469 |  | 0.797 (0.693, 0.917) | 1.49E-03 | 1.73E-03 | 1.820 |
|  | High | 0.433 (0.351, 0.535) | 8.24E-15 | 1.74E-14 | 4.048 |  | 0.705 (0.564, 0.881) | 2.15E-03 | 2.42E-03 | 2.189 |

Abbreviations: SCORE2, Systemic coronary risk estimation 2; LE’8, Life’s essential 8; HLS, healthy life scores; ELSA, the English Longitudinal Study of Ageing; HRS, the Health and Retirement Study; FDR, false discovery rate.

Effect estimates were hazard ratio and 95%-confidence interval derived from Cox regression model, which estimated the associations between different lifestyle scores and multimorbidity. The crude model adjusted nothing.

^a^ SCORE2 has been classically categorized in low (1-4%), medium (5-9%), high (>10%) risk categories. The higher the score of SCORE2, the more dangerous it was. ^b^ LE’8 has been classically categorized in low (0-49), medium (50-79), high (>79) categories. HLS has been classically categorized in low (0-1), medium (2-3), high (4-5) categories. The LE’8 and HLS were reversed, with higher scores showing healthier.

**eTable 4. Simple Slopes between different lifestyle scores and multimorbidity in two cohorts.**

| Scoring models | ELSA | | HRS | |
| --- | --- | --- | --- | --- |
|  | β (95% *CI*) | *P* value | β (95% *CI*) | *P* value |
| SCORE2 ^a^ | 0.052 (0.043, 0.060) | <0.001 | 0.033 (0.026, 0.040) | <0.001 |
| LE'8 ^b^ | -0.007 (-0.009, -0.006) | <0.001 | -0.008 (-0.010, -0.006) | <0.001 |
| HLS ^b^ | -0.099 (-0.117, -0.080) | <0.001 | -0.041 (-0.065, -0.018) | <0.001 |

Abbreviations: SCORE2, Systemic coronary risk estimation 2; LE’8, Life’s essential 8; HLS, healthy life scores; ELSA, the English Longitudinal Study of Ageing; HRS, the Health and Retirement Study.

^a^ The higher the score of SCORE2, the more dangerous it was.

^b^ The LE’8 and HLS were reversed, with higher scores showing healthier.

**eTable 5. The differentiation and calibration of SCORE2 prediction model in updated datasets in two cohorts.**

| SCORE2 model | ELSA | | HRS | |
| --- | --- | --- | --- | --- |
|  | Male | Female | Male | Female |
| Calibration slope | 0 | 0 | 0 | 0 |
| Calibration intercept | -0.6811 | -0.9655 | 0.2642 | -0.1227 |
| AIC | 1283 | 1488 | 783.1 | 921.5 |
| AUC (95% CI) | 0.649 (0.613, 0.684) | 0.696 (0.665, 0.728) | 0.657 (0.611, 0.702) | 0.667 (0.627, 0.708) |

Abbreviations: SCORE2, Systemic coronary risk estimation 2; AIC, Akaike information criterion; AUC, Area Under Curve; ELSA, the English Longitudinal Study of Ageing; HRS, the Health and Retirement Study.

**eTable 6. Associations of different lifestyle scores with multimorbidity (＞2 years) in two cohorts.**

| Scoring models | | ELSA | | | | |  | HRS | | | |
| --- | --- | --- | --- | --- | --- | --- | --- | --- | --- | --- | --- |
|  | | | Crude | *P* value | Adjusted | *P* value |  | Crude | *P* value | Adjusted | *P* value |
| SCORE2 ^a^ | Per one-point increment | | 1.250 (1.189, 1.314) | < 2e-16 | 1.236 (1.176, 1.300) | < 2e-16 |  | 1.072 (1.044, 1.102) | 3.70E-07 | 1.074 (1.044, 1.104) | 5.38E-07 |
|  | Low | | Reference |  | Reference |  |  | Reference |  | Reference |  |
|  | Medium | | 2.465 (1.873, 3.245) | 1.23E-10 | 2.394 (1.818, 3.152) | 5.11E-10 |  | 1.976 (1.356, 2.881) | 3.97E-04 | 1.934 (1.326, 2.820) | 6.14E-04 |
|  | High | | 5.624 (3.145, 10.056) | 5.71E-09 | 5.086 (2.840, 9.110) | 4.50E-08 |  | 2.711 (1.816, 4.047) | 1.08E-06 | 2.641 (1.765, 3.951) | 2.32E-06 |
| LE'8 ^b^ | Per one-point increment | | 0.959 (0.950, 0.968) | <2e-16 | 0.962 (0.952, 0.972) | 3.42E-14 |  | 0.975 (0.964, 0.986) | 5.99E-06 | 0.976 (0.965, 0.987) | 3.61E-05 |
|  | Low | | Reference |  | Reference |  |  | Reference |  | Reference |  |
|  | Medium | | 0.409 (0.321, 0.521) | 4.85E-13 | 0.437 (0.342, 0.558) | 3.37E-11 |  | 0.739 (0.458, 1.192) | 2.15E-01 | 0.690 (0.427, 1.114) | 1.29E-01 |
|  | High | | 0.138 (0.034, 0.558) | 5.46E-03 | 0.194 (0.048, 0.785) | 2.15E-02 |  | 0.366 (0.200, 0.671) | 1.15E-03 | 0.396 (0.215, 0.728) | 2.89E-03 |
| HLS ^b^ | Per one-point increment | | 0.720 (0.639, 0.811) | 6.04E-08 | 0.699 (0.620, 0.789) | 5.65E-09 |  | 0.940 (0.833, 1.060) | 3.12E-01 | 0.948 (0.841, 1.068) | 3.81E-01 |
|  | Low | | Reference |  | Reference |  |  | Reference |  | Reference |  |
|  | Medium | | 0.766 (0.503, 1.168) | 2.16E-01 | 0.713 (0.467, 1.088) | 1.16E-01 |  | 0.798 (0.576, 1.105) | 1.74E-01 | 0.800 (0.577, 1.109) | 1.81E-01 |
|  | High | | 0.399 (0.247, 0.644) | 1.66E-04 | 0.358 (0.222, 0.579) | 2.73E-05 |  | 0.998 (0.657, 1.517) | 9.93E-01 | 1.037 (0.682, 1.578) | 8.64E-01 |

Abbreviations: SCORE2, Systemic coronary risk estimation 2; LE’8, Life’s essential 8; HLS, healthy life scores; ELSA, the English Longitudinal Study of Ageing; HRS, the Health and Retirement Study.

Effect estimates were hazard ratio and 95%-confidence interval derived from Cox regression model, which estimated the associations between health behavior score and multimorbidity. Participants had suffered from multimorbidity for > 2 years.

^a^ SCORE2 has been classically categorized in low (1-4%), medium (5-9%), high (>10%) risk categories. The higher the score of SCORE2, the more dangerous it was.

^b^ LE’8 has been classically categorized in low (0-49), medium (50-79), high (>79) categories. HLS has been classically categorized in low (0-1), medium (2-3), high (4-5) categories. The LE’8 and HLS were reversed, with higher scores showing healthier.

The crude model adjusted nothing. SCORE2 model was adjusted for drinking, race, and BMI; LE’8 model was adjusted for age, sex, drinking, and race; HLS model was adjusted for age, sex, and race.

**eTable 7. Associations of different lifestyle scores with multimorbidity by using Inverse probability weighting in two cohorts.**

| Scoring models |  | ELSA | |  | HRS | |
| --- | --- | --- | --- | --- | --- | --- |
|  |  | *HR* (95% *CI*) | *P* value |  | *HR* (95% *CI*) | *P* value |
| SCORE2 ^a^ | Per one-point increment | 1.221 (1.187, 1.255) | < 2e-16 |  | 1.084 (1.068, 1.101) | < 2e-16 |
|  | Low | Reference |  |  | Reference |  |
|  | Medium | 2.085 (1.785, 2.436) | < 2e-16 |  | 1.700 (1.386, 2.085) | 3.64E-07 |
|  | High | 4.252 (3.023, 5.982) | < 2e-16 |  | 2.830 (2.301, 3.481) | < 2e-16 |
| LE’8 ^b^ | Per one-point increment | 0.973 (0.967, 0.979) | < 2e-16 |  | 0.981 (0.976, 0.986) | 2.62E-13 |
|  | Low | Reference |  |  | Reference |  |
|  | Medium | 0.596 (0.524, 0.678) | 4.20E-15 |  | 0.730 (0.596, 0.893) | 2.22E-03 |
|  | High | 0.753 (0.395, 1.437) | 3.90E-01 |  | 0.505 (0.380, 0.671) | 2.35E-06 |
| HLS ^b^ | Per one-point increment | 0.733 (0.689, 0.779) | < 2e-16 |  | 0.905 (0.856, 0.956) | 3.75E-04 |
|  | Low | Reference |  |  | Reference |  |
|  | Medium | 0.580 (0.471, 0.714) | 2.68E-07 |  | 0.839 (0.727, 0.967) | 1.55E-02 |
|  | High | 0.381 (0.302, 0.479) | < 2e-16 |  | 0.836 (0.675, 1.035) | 9.93E-02 |

Abbreviations: HR, hazard ratio; CI, confidence interval; SCORE2, Systemic coronary risk estimation 2; LE’8, Life’s essential 8; HLS, healthy life scores; ELSA, the English Longitudinal Study of Ageing; HRS, the Health and Retirement Study.

Effect estimates were hazard ratio and 95%-confidence interval derived from Cox regression model.

^a^ SCORE2 has been classically categorized in low (1-4%), medium (5-9%), high (>10%) risk categories. The higher the score of SCORE2, the more dangerous it was. ^b^ LE’8 has been classically categorized in low (0-49), medium (50-79), high (>79) categories. HLS has been classically categorized in low (0-1), medium (2-3), high (4-5) categories. The LE’8 and HLS were reversed, with higher scores showing healthier.

SCORE2 model was adjusted for drinking, race, and BMI; LE’8 model was adjusted for age, sex, drinking, and race; HLS model was adjusted for age, sex, and race.

**eTable 8. Comparison of the risk of different lifestyle scores with multimorbidity in the original Cox model vs. the competing-risks regression model in the HRS.**

| Scoring models | Cox model | |  | Competing-risks model | |
| --- | --- | --- | --- | --- | --- |
|  | *HR* (95% *CI*) | *P* value |  | *HR* (95% *CI*) | *P* value |
| SCORE2 ^a^ | 1.085 (1.070, 1.101) | <2e-16 |  | 1.633 (1.487, 1.792) | 0.00E+00 |
| LE'8 ^b^ | 0.981 (0.976, 0.986) | 1.04E-12 |  | 0.687 (0.613, 0.770) | 9.1E-11 |
| HLS ^b^ | 0.897 (0.847, 0.950) | 1.91E-04 |  | 0.877 (0.800, 0.962) | 5.3E-03 |

Abbreviations: HR, hazard ratio; CI, confidence interval; SCORE2, Systemic coronary risk estimation 2; LE’8, Life’s essential 8; HLS, healthy life scores.

Effect estimates were hazard ratio and 95%-confidence interval derived from Cox regression model and competing-risks model. SCORE2 model was adjusted for drinking, race, and BMI; LE’8 model was adjusted for age, sex, drinking, and race; HLS model was adjusted for age, sex, and race.

^a^ The higher the score of SCORE2, the more dangerous it was.

^b^ The LE’8 and HLS were reversed, with higher scores showing healthier.


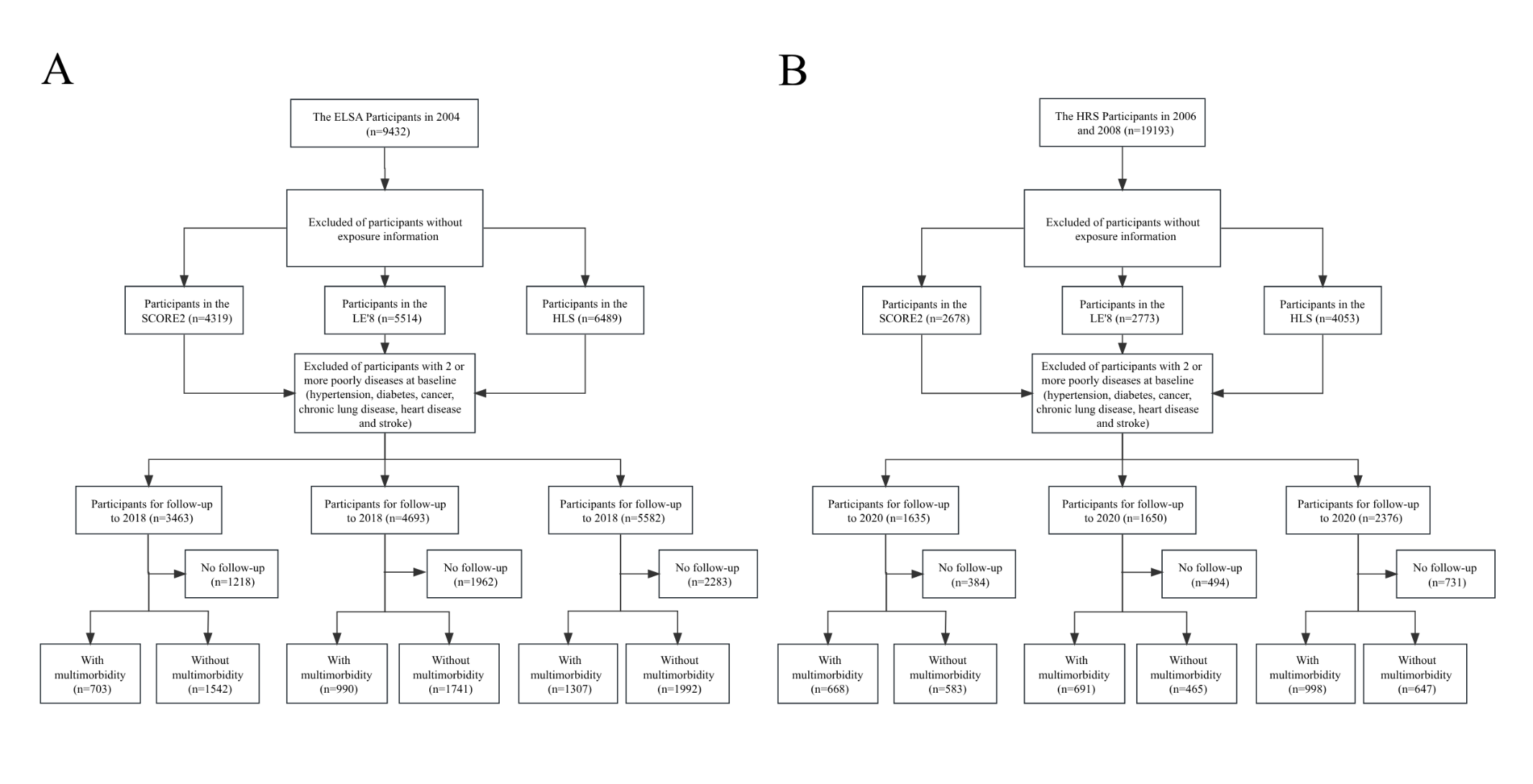


**eFigure 1.** Flowchart of the participants selection. A in the English Longitudinal Study of Ageing (ELSA); B in the Health and Retirement Study (HRS).

**
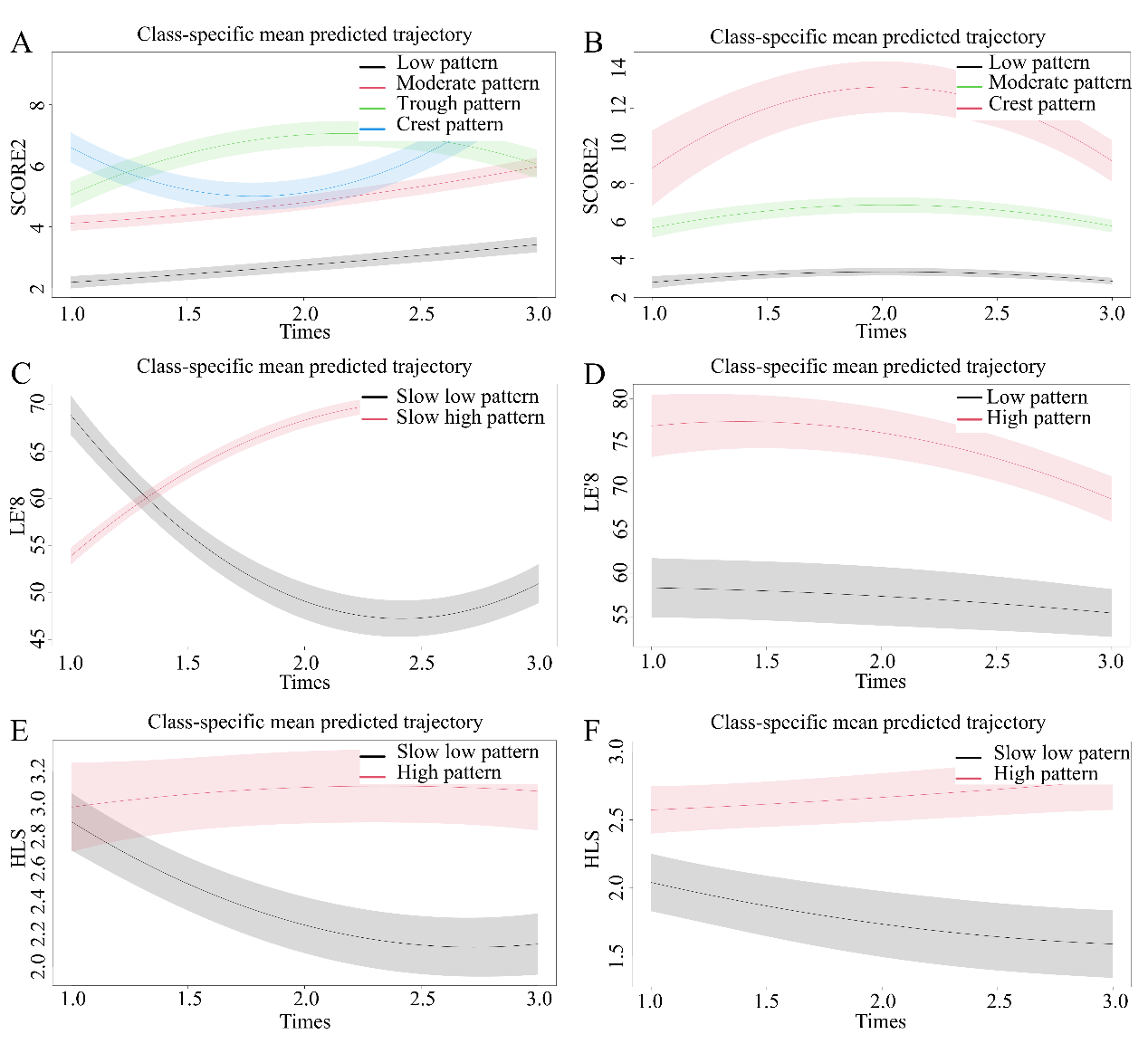
**

**eFigure 2. The trajectory grouping results of different lifestyle scores and risk of multimorbidity in two cohorts.** A-B. the trajectory grouping results of SCORE2 in the ELSA, and in the HRS. C-D. the trajectory grouping results of LE’8 in the ELSA, and in the HRS. E-F. the trajectory grouping results of HLS in the in the ELSA, and in the HRS. Abbreviations: SCORE2, Systemic coronary risk estimation 2; LE’8, Life’s essential 8; HLS, healthy life scores; ELSA, English Longitudinal Study of Ageing; HRS, Health and Retirement Study.

**
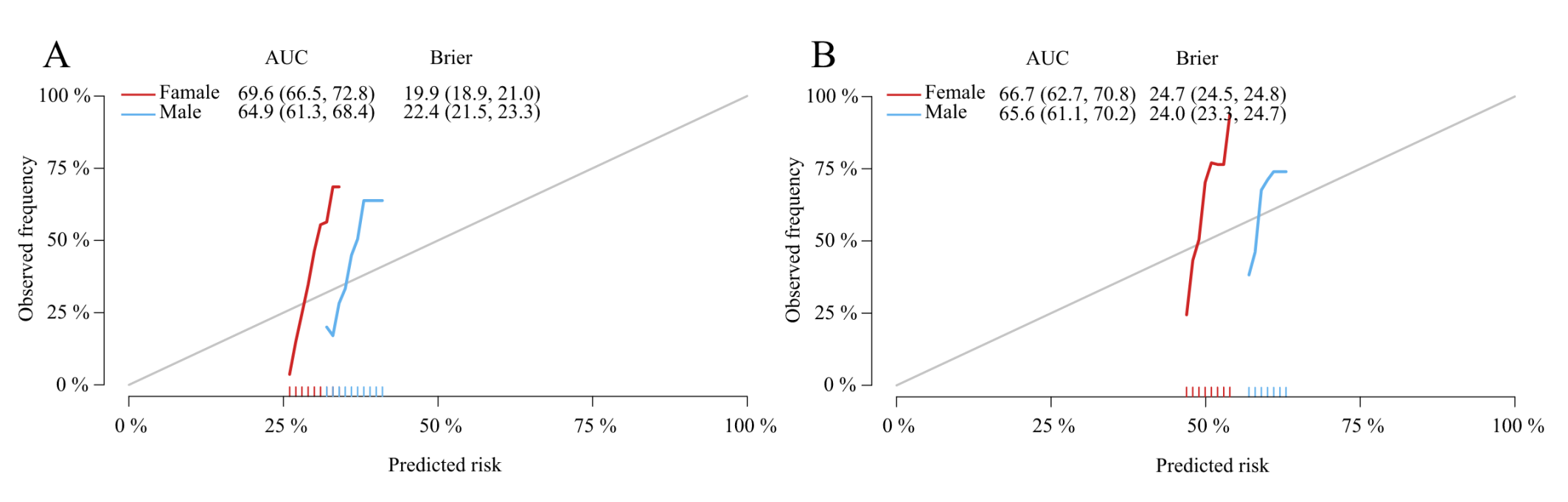
**

**eFigure 3. The differentiation and calibration of SCORE2 prediction model in updated datasets in two cohorts.** A. in the English Longitudinal Study of Ageing (ELSA), B. in the Health and Retirement Study (HRS).
